# Construction of brain metastasis prediction model in limited stage small cell lung cancer patients without prophylactic cranial irradiation

**DOI:** 10.1111/crj.13730

**Published:** 2024-01-15

**Authors:** Jiayi Guo, Jianjiang Liu, Wanli Ye, Jun Xu, Wangyan Zhong, Xiaoyu Zhang, Hang Yuan, Hao Shi, Ting Li, Yibing Xu, Jiwei Mao, Bin Shen, Dongping Wu

**Affiliations:** ^1^ School of Medicine Shaoxing University Shaoxing Zhejiang China; ^2^ Department of Radiation Oncology First Affiliated Hospital of Shaoxing University Shaoxing Zhejiang China; ^3^ Shaoxing People's Hospital Shaoxing Zhejiang China

**Keywords:** brain metastasis, limited stage, nomogram, prophylactic cranial irradiation, small cell lung cancer

## Abstract

**Introduction:**

Small cell lung cancer (SCLC) is a highly aggressive lung cancer variant known for its elevated risk of brain metastases (BM). While earlier meta‐analyses supported the use of prophylactic cranial irradiation (PCI) to reduce BM incidence and enhance overall survival, modern MRI capabilities raise questions about PCI's universal benefit for limited‐stage SCLC (LS‐SCLC) patients. As a response, we have created a predictive model for BM, aiming to identify low‐risk individuals who may not require PCI.

**Methods:**

A total of 194 LS‐SCLC patients without PCI treated between 2009 and 2021 were included. We conducted both univariate and multivariate analyses to pinpoint the factors associated with the development of BM. A nomogram for predicting the 2‐ and 3‐year probabilities of BM was then constructed.

**Results:**

Univariate and multivariate analyses revealed several significant independent risk factors for the development of BM. These factors include TNM stage, the number of chemotherapy (ChT) cycles, Ki‐67 expression level, pretreatment serum lactate dehydrogenase (LDH) levels, and haemoglobin (HGB) levels. These findings underscore their respective roles as independent predictors of BM. Based on the results of the final multivariable analysis, a nomogram model was created. In the training cohort, the nomogram yielded an area under the receiver operating characteristic curve (AUC) of 0.870 at 2 years and 0.828 at 3 years. In the validation cohort, the AUC values were 0.897 at 2 years and 0.789 at 3 years. The calibration curve demonstrated good agreement between the predicted and observed probabilities of BM.

**Conclusions:**

A novel nomogram has been developed to forecast the likelihood of BM in patients diagnosed with LS‐SCLC. This tool holds the potential to assist healthcare professionals in formulating more informed and tailored treatment plans.

## Graphical abstract

A novel nomogram was constructed for predicting the risk of BM in LS‐SCLC patients, which could help clinicians make better treatment strategies.

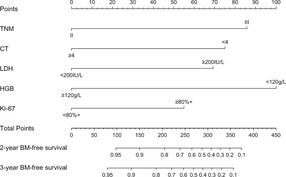

## 1 INTRODUCTION

Small cell lung cancer (SCLC) is a highly malignant tumour exposed to a very high level of risk of brain metastases (BM), found in at least 15% of patients at the time of initial diagnosis. The rate may increase up to 24–33% if pre‐treatment staging is performed using cranial magnetic resonance imaging (MRI),^1^, ^2^ and another 40–50% of patients will develop BM at a later time.^3^, ^4^

A prior meta‐analysis provided confirmation^5^ that prophylactic cranial irradiation (PCI) contributed to a reduction in the incidence of BM, diminishing it from 58.6% to 33.3%. Furthermore, it yielded a notable 3‐year absolute increase in overall survival (OS) rates, amounting to 5.4% for patients with SCLC, particularly those with limited‐stage disease. However, it is crucial to note that the majority of the studies included in this analysis were conducted during a period when advanced imaging techniques like positron emission tomography with 18F‐fluorodeoxyglucose (18F‐FDG PET) and cranial MRI were not universally accessible. Additionally, stringent follow‐up protocols involving cranial MRI were not consistently applied. These factors may have had a considerable impact on the initial staging accuracy and the eventual survival outcomes of the patients involved. On the one hand, due to the inability of ordinary cranial computed tomography (CT) to detect small BM, patients who have not undergo pretreatment cranial MRI or only underwent pretreatment cranial CT may receive ‘therapeutic rather than preventive PCI’, which will strengthen the role of PCI. On the other hand, due to the lack of strict monitoring of cranial MRI, patients who had not undergone PCI might miss the opportunity for ‘salvage PCI’ (25‐30Gy/10F/DT) to improve OS. These above made the strategy of improving OS in SCLC by using PCI rather controversial.

While PCI has demonstrated its capacity to lower the occurrence of BM, it remains burdened with several drawbacks. First, it impairs their cognitive function^6^, ^7^ and increases the financial burden of patients; second, even if BM appears in some patients, it is caused by uncontrolled extracranial tumours leading to subsequent metastases to the brain, rather than from the beginning. Thus, for these patients, PCI at the beginning may not reduce the occurrence of BM; third, some studies suggest that the incidence of BM remains 4% at the first year, 30% at the second year, 11.2–38% at the third year and 44% at the fourth year, even after PCI^8^, ^9^, ^10^, ^11^ and that it is difficult to increase the dose of palliative radiotherapy in patients who have previously undergone PCI after the recurrence of BM, while the adverse effects significantly increased.

Therefore, identifying low‐risk patients with BM and enabling them to avoid PCI is of great significance. We hope this study can provide some inspiration for solving this dilemma.

## 2 MATERIALS AND METHODS

In this retrospective analysis, a cohort of 235 consecutive patients diagnosed with limited‐stage small cell lung cancer (LS‐SCLC) who did not undergo PCI was included. These patients underwent treatment at XXX Hospital between January 2009 and December 2021. The staging of these individuals was determined in accordance with the 7th edition of the Alternate Joint Communication Center (AJCC) Lung Cancer Staging criteria. In principle, the preferred first‐line treatment for patients with limited‐stage SCLC enrolled in our study was still platinum‐based concurrent chemoradiotherapy, and the recommended number of cycles of chemotherapy is 4–6. Moreover, they underwent a comprehensive diagnostic assessment that included cranial MRI, contrast‐enhanced chest CT, full abdominal CT with contrast (when applicable), or full abdominal MRI, cervical MRI, and 18F‐FDG‐PET (preferred) or positron emission tomography. All of them were tested for serum lactate dehydrogenase (LDH), platelet (PLT), haemoglobin (HGB), carcinoembryonic antigen (CEA) and neuron‐specific enolase (NSE) level before treatment, and the cut‐off values for normal and high levels of LDH, PLT, HGB, CEA and NSE in this study were 200 IU/L, 280*10^9^/L, 120 g/L, 10 and 15.4 ng/ml, respectively. In addition, all patients underwent immunohistochemistry for the tumour markers Ki‐67 and TTF‐1. We employed the Response Evaluation Criteria in Solid Tumors (RECIST) version 1.1 criteria to evaluate the short‐term treatment efficacy in all the patients.^12^

**FIGURE 1:**
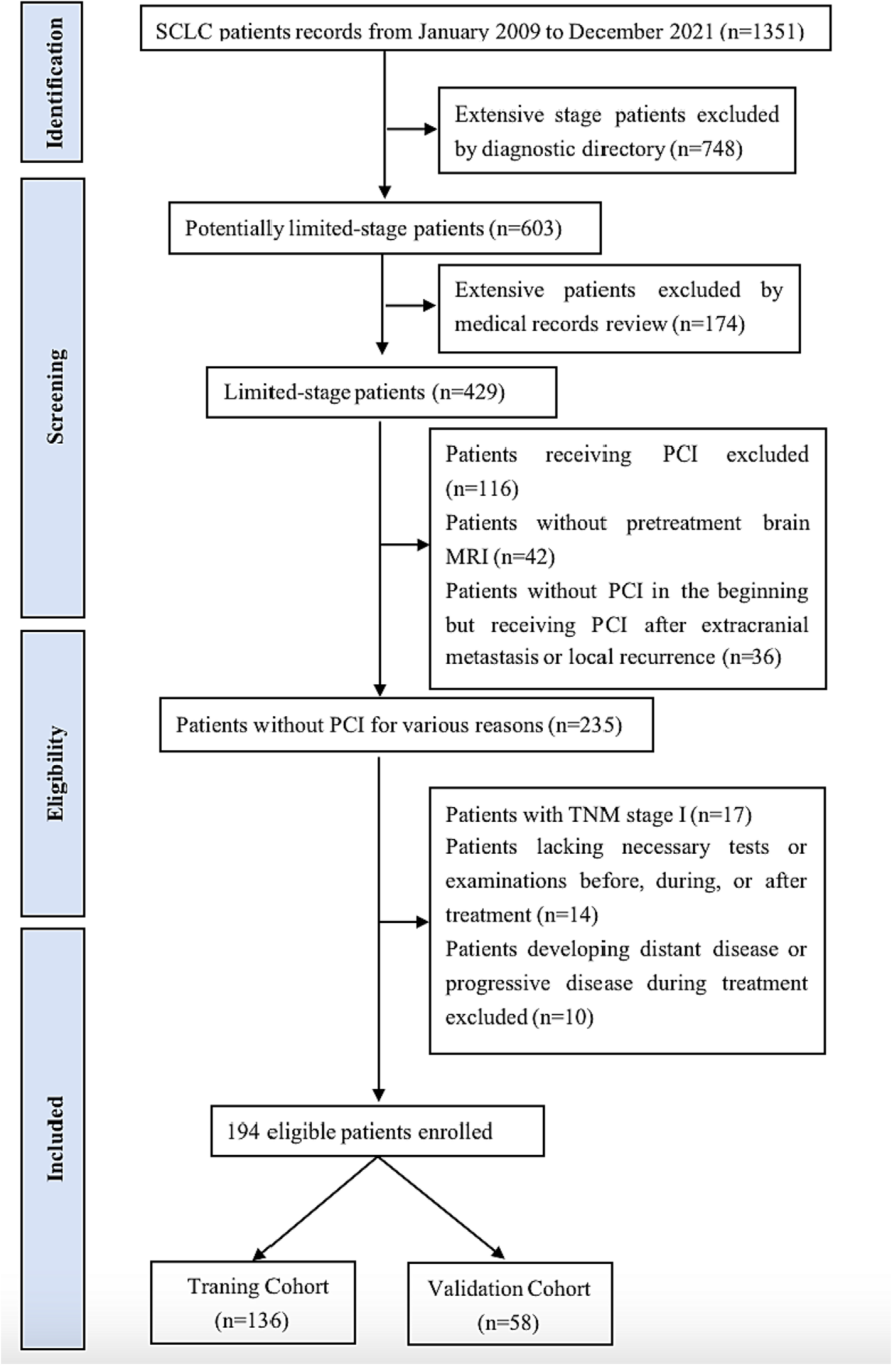
Flow chart of patient enrollment.

From the initial group of 235 patients, we excluded 17 individuals with TNM Stage I, 14 who lacked essential before, during, or after treatment assessments and 10 who exhibited distant metastases or progressive disease during treatment. As a result, 194 patients met the rigorous eligibility criteria for our analysis. These patients were then randomly divided into two separate cohorts: a training cohort, which comprised 136 patients, and a validation cohort, consisting of 58 patients. The detailed breakdown of this enrollment process is provided in Figure 1.

Patients underwent cranial MRI (preferred) or brain CT every 3–4 months for 1 year after the end of treatment and every 6 months from the second year onwards. During the follow‐up, all patients were evaluated with a blood test, a CT scan of the chest, a CT scan of the abdomen or a B‐ultrasound and other examinations as required by their symptoms.

The primary endpoint is the incidence of BM, specifically intracranial recurrence identified via MRI or CT scans conducted throughout the entire disease progression. The secondary endpoint is OS, calculated from the initial diagnosis date until either the date of death or the last follow‐up.

## 3 STATISTICAL ANALYSIS

We conducted statistical analyses using R software (version 4.0.3). Categorical variables are presented as numbers and percentages and were assessed using either the χ2 test or Fisher's exact test. To gauge the strength of the association, we utilized the hazard ratio (HR) along with a 95% confidence interval (CI). Factors with a significance level of *p* < 0.10 were incorporated into the multivariate Cox regression analysis.

We evaluated the clinical utility and predictive capacity of a prediction model based on independent risk factors. The discriminatory capability of the multivariate Cox model in predicting the likelihood of BM at both 2‐ and 3‐year intervals was assessed using measures akin to the area under the receiver operating characteristic (ROC) curve.

To evaluate the model's predictive accuracy, we generated calibration curves. Furthermore, we utilized decision curve analysis (DCA) to assess the practical clinical value of the nomogram model by quantifying the net benefit across different threshold probabilities.

## 4 RESULTS

### 4.1 Patient characteristics

Table 1 provides an overview of patient characteristics from two distinct groups: the No Brain Metastasis (No BM) group, consisting of 137 patients, and the BM group, consisting of 57 patients. The overall incidence of BM was 29.4% (57 out of 194 patients), and the median time to intracranial failure was 12.7 months, with a range of 1.37 to 39.2 months.

**TABLE 1:** Summary of patients clinical and pathological characteristic.

Variable	Total (*n* = 194)	No BM (*n* = 137)	BM (*n* = 57)	Statistic	P
Gender, *n* (%)				χ^2^ = 2.206	0.137
Male	135 (69.59)	91 (66.42)	44 (77.19)		
Female	59 (30.41)	46 (33.58)	13 (22.81)		
Age, *n* (%)				χ^2^ = 7.086	0.008
<60	44 (22.68)	24 (17.52)	20 (35.09)		
≥60	150 (77.32)	113 (82.48)	37 (64.91)		
Smoking index, *n* (%)				χ^2^ = 0.411	0.814
0	76 (39.18)	52 (37.96)	24 (42.11)		
1–800	78 (40.21)	57 (41.61)	21 (36.84)		
>800	40 (20.62)	28 (20.44)	12 (21.05)		
ECOG‐PS, *n* (%)				χ^2^ = 2.418	0.120
0–1	168 (86.6)	122 (89.05)	46 (80.70)		
≥2	26 (13.4)	15 (10.95)	11 (19.30)		
TNM stage, *n* (%)				χ^2^ = 13.221	<0.001
II	56 (28.87)	50 (33.50)	6 (10.53)		
III	138 (71.13)	87 (63.50)	51 (89.47)		
ChT cycles, *n* (%)				χ^2^ = 0.635	0.426
<4	121 (60.82)	83 (60.58)	38(66.67)		
≥4	73 (37.63)	54 (39.42)	19 (33.33)		
Radiotherapy, *n* (%)				χ^2^ = 7.529	0.006
No	111 (57.22)	87 (63.50)	24 (42.11)		
Yes	83 (42.78)	50 (36.50)	33 (57.89)		
Concurrent ChT, *n* (%)				χ^2^ = 2.377	0.123
No	147 (75.77)	108 (78.83)	39 (68.42)		
Yes	47 (24.23)	29 (21.17)	18 (31.58)		
Treatment Response, *n* (%)				χ^2^ = 6.948	0.008
CR	68 (35.05)	56 (40.88)	12 (21.05)		
No CR	126 (64.95)	81 (59.12)	45 (78.95)		
TTF‐1, *n* (%)				χ^2^ = 2.222	0.136
−	60 (30.93)	38(27.74)	22 (38.60)		
+	134 (69.07)	99 (72.26)	35 (61.40)		
Ki‐67, *n* (%)				χ^2^ = 5318	0.021
<80+	86 (44.33)	68 (49.64)	18 (31.58)		
≥80+	108 (55.67)	69 (50.36)	39 (68.42)		
Pretreatment CEA, *n* (%)				χ^2^ = 3.598	0.058
<10 ng/ml	138 (71.13)	92 (67.15)	46 (80.70)		
≥10 ng/ml	56 (28.87)	45 (32.85)	11 (19.30)		
Pretreatment HGB, *n* (%)				χ^2^ = 12.480	<0.001
>120 g/L	96 (49.48)	79 (56.77)	17 (29.82)		
≤120 g/l	98 (50.52)	58 (42.43)	40 (70.18)		
Pretreatment PLT, *n* (%)				χ^2^ = 5.203	0.023
<280*109/L	64 (32.99)	52 (37.96)	12 (21.05)		
≥280*109/L	130 (67.01)	85 (62.04)	45 (78.95)		
Pretreatment LDH, *n* (%)				χ^2^ = 11.215	<0.001
<200 IU/l	94 (48.45)	77 (54.74)	17 (29.82)		
≥200 IU/l	100 (51.55)	60 (45.26)	40 (70.18)		
Pretreatment NSE, *n* (%)				χ^2^ = 11.949	<0.001
<15.4 ng/ml	153 (78.87)	117 (85.40)	36 (63.16)		
≥15.4 ng/ml	41 (21.13)	20 (14.60)	21 (36.84)		^i^

Abbreviations: CEA, carcinoembryonic antigen; ChT, chemotherapy; CR, complete response; ECOG‐PS, Eastern Cooperative Oncology Group performance status; HGB, haemoglobin; LDH, serum lactate dehydrogenase; No BM, no appearance of brain metastasis; NSE, neuron specific enolase; P, Pearson's χ^2^ test was used to calculate the *p* value; PLT, platelet; Smoking Index, number of cigarettes smoked per day × years of smoking; TTF‐1, thyroid transcription factor‐1.

The median age of the entire patient population was 67 years, ranging from 45 to 88 years. Specifically, patients in the No BM group had a median age of 68 years, ranging from 45 to 88 years, while those in the BM group had a median age of 64 years, ranging from 45 to 81 years.

It is important to highlight that, upon random division of the 194 patients into two cohorts, namely, a training cohort comprising 136 patients and a validation cohort comprising 58 patients (as detailed in Table S1), there were no statistically significant differences observed in any of the factors. This division was made to facilitate the analysis while ensuring that both cohorts remained comparable in terms of their characteristics.

### 4.2 Cumulative incidence rate of BM

The median follow‐up time was 19.1 months (range: 3.0–142.8 months) for all patients, 21.9 months (range: 3.0–142.8) for the BM‐free group and 17.2 months (range: 4.6–51.6) for the BM group. As shown in Table 2, according to univariate analysis, patients in the training cohort with TNM stage III, no complete response (CR), chemotherapy cycles (ChT cycles) < 4, Ki‐67 ≥ 80%+, pretreatment LDH ≥ 200 IU/L, pretreatment NSE ≥ 15.4 ng/ml and pretreatment HGB < 120 g/L were more likely to develop BM. In our multivariable analysis, we determined that several factors independently contribute to the risk of developing BM: patients with TNM Stage III, fewer than four chemotherapy cycles, high Ki‐67 expression (≥80%+), elevated pretreatment LDH (≥200 IU/L) and lower pretreatment HGB (<120 g/L) were all associated with an increased risk of BM in the studied population.

**TABLE 2:** Univariate and multivariate analysis predicting the risk of BM in patients without prophylactic cranial irradiation (PCI) in the training cohort.

Variables	Univariate analysis			Multivariate analysis		
	HR	95% CI	*P*	HR	95% CI	*P*
TNM Stage (III vs. II)	4.32	1.68–11.10	0.002	2.90	1.03–8.12	0.043
ChT cycles (<4 vs. ≥4)	2.57	1.13–5.81	0.024	2.56	1.03–6.38	0.043
Ki‐67 (≥80% + vs. <80%+)	2.13	1.13–3.99	0.019	1.97	1.03–3.75	0.040
Pretreatment LDH (≥200 IU/L vs. <200 IU/L)	2.97	1.48–5.96	0.002	2.63	1.26–5.49	0.010
Pretreatment HGB (≥120 g/L vs. <120 g/L)	0.40	0.19–0.84	0.016	0.36	0.14–0.96	0.041
Treatment Response (No CR vs. CR)	4.32	1.04–17.93	0.044	2.23	0.39–12.70	0.366
Pretreatment CEA (≥10 ng/ml vs. <10 ng/ml)	2.50	0.90–7.41	0.079	1.17	0.25–5.35	0.838
Pretreatment NSE (≥15.4 ng/ml vs. <15.4 ng/ml)	3.05	1.62–5.73	0.001	1.23	0.61–2.48	0.561
Pretreatment PLT (≥280*109/L vs. <280*109/L)	0.51	0.24–1.06	0.072	0.65	0.30–1.40	0.274
Gender (male vs. female)	0.85	0.43–1.67	0.628			
Age (≥60 vs. <60)	1.57	0.82–3.02	0.173			
**Smoking index**						
(1–800 vs. 0)	0.75	0.37–1.51	0.421			
(>800 vs. 0)	0.72	0.31–1.71	0.459			
ECOG PS (0–1 vs. ≥2)	1.28	0.54–3.05	0.578			
Radiotherapy (Yes vs. NO)	1.42	0.76–2.66	0.276			
Concurrent ChT (Yes vs. No)	1.11	0.55–2.22	0.768			
TTF‐1 (+ vs. −)	1.66	0.87–3.16	0.122			

Abbreviations: CI, confidence interval; HR, hazard ratio.

### 4.3 Nomogram construction and evaluation

**FIGURE 2:**
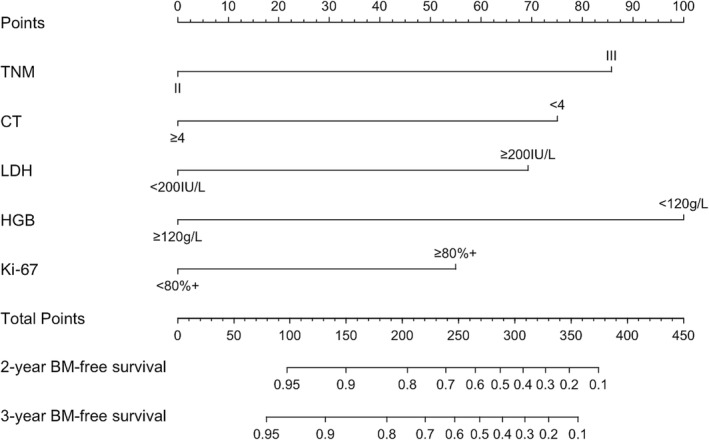
Nomogram to predict the BM‐free survival in limited‐stage small‐cell lung cancer (LS‐SCLC) patients without PCI in the training cohort.

**FIGURE 3:**
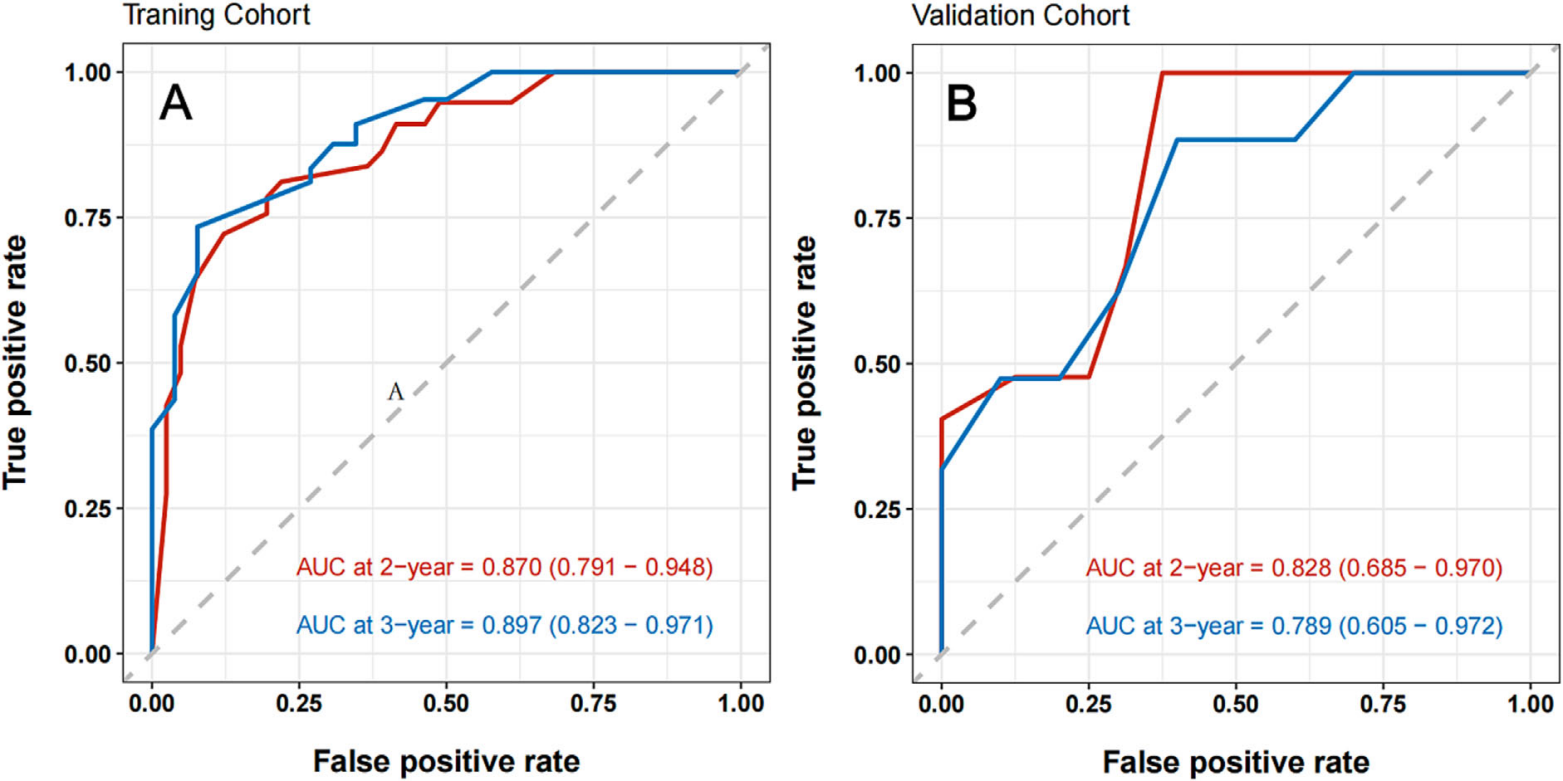
Receiver operating characteristics (ROC) curve and area under the ROC (AUC) in the training (A) and validation cohorts (B).

**FIGURE 4:**
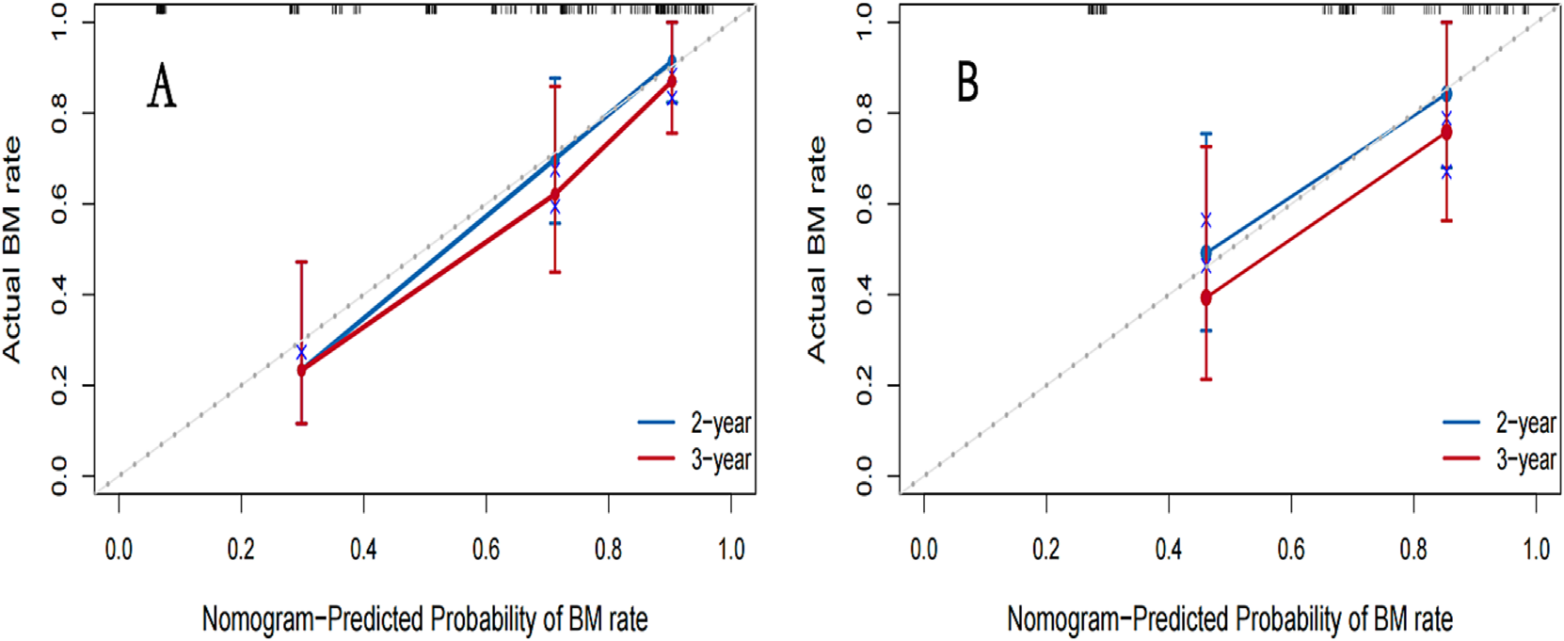
Calibration curves of the nomogram prediction in the training (A) and validation cohorts (B).

**FIGURE 5:**
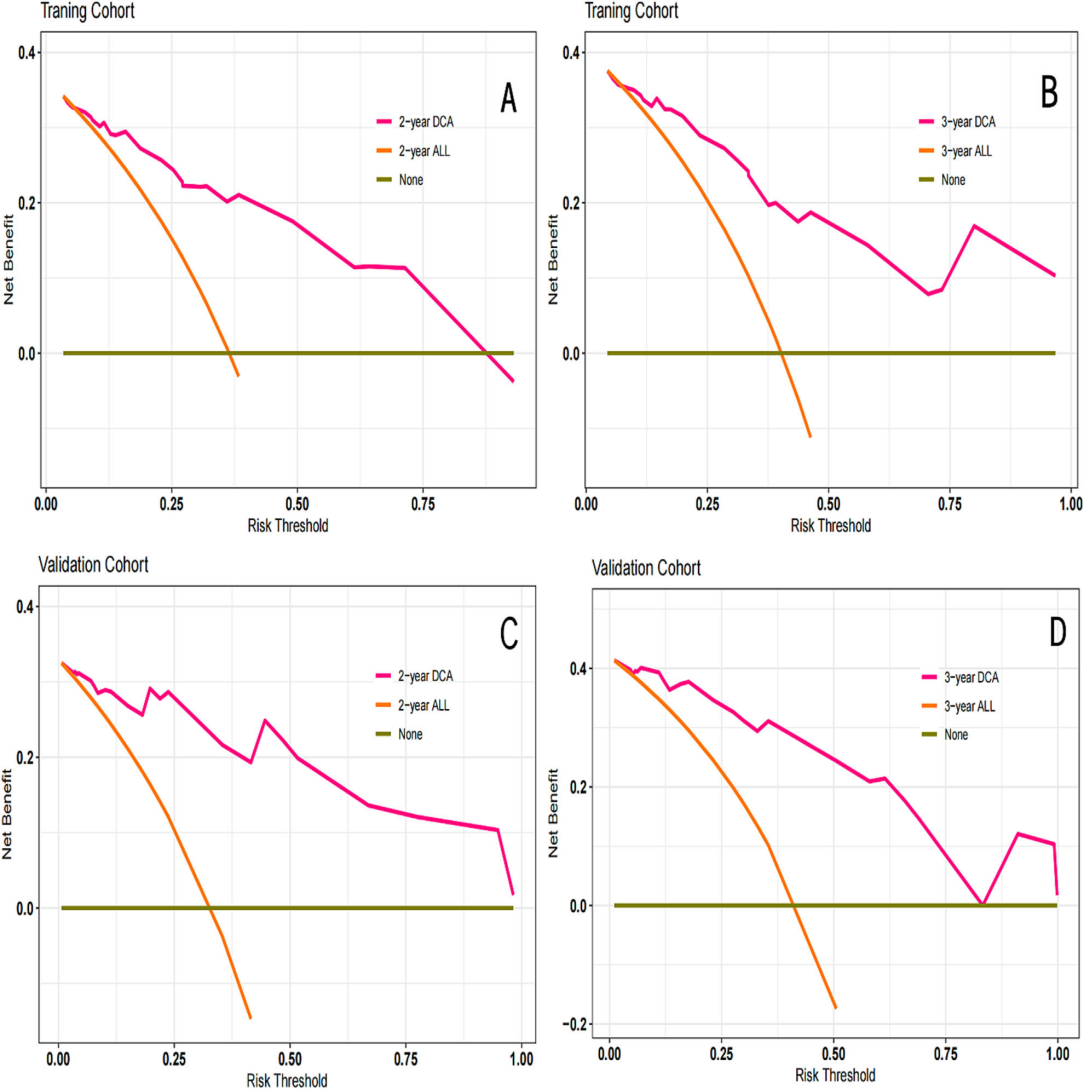
Decision curve analysis (DCA) at 2 and 3 years for the nomogram in the training (A, B) and validation cohorts (C, D).

To identify variables associated with brain metastasis‐free survival in the training cohort, univariate and multivariate COX regression analyses were perform. The results are shown in Table 2. On the basis of these factors, a predictive nomogram was constructed to predict the 2‐ and 3‐year accumulated incidence of BM in patients with LS‐SCLC in the training cohort (Figure 2). Moreover, when evaluating the predictive capabilities of the Nomogram, we noted that the area under the receiver operating characteristic curve (AUC) was 0.870 at 2 years and 0.897 at 3 years for the training cohort, while for the validation cohort, the AUC was 0.828 at 2 years and 0.789 at 3 years, as depicted in Figure 3. In both the training and validation cohorts, the nomogram's calibration curve, assessing the probability of BM at 2 and 3 years, demonstrated excellent alignment between the nomogram's predictions and actual observations, as illustrated in Figure 4. Furthermore, the results of the DCA demonstrate that the utilization of the nomogram for predicting BM in LS‐SCLC patients would be beneficial when the threshold probability aligns with the appropriate range, as depicted in Figure 5.

### 4.4 BM risk stratification

**FIGURE 6:**
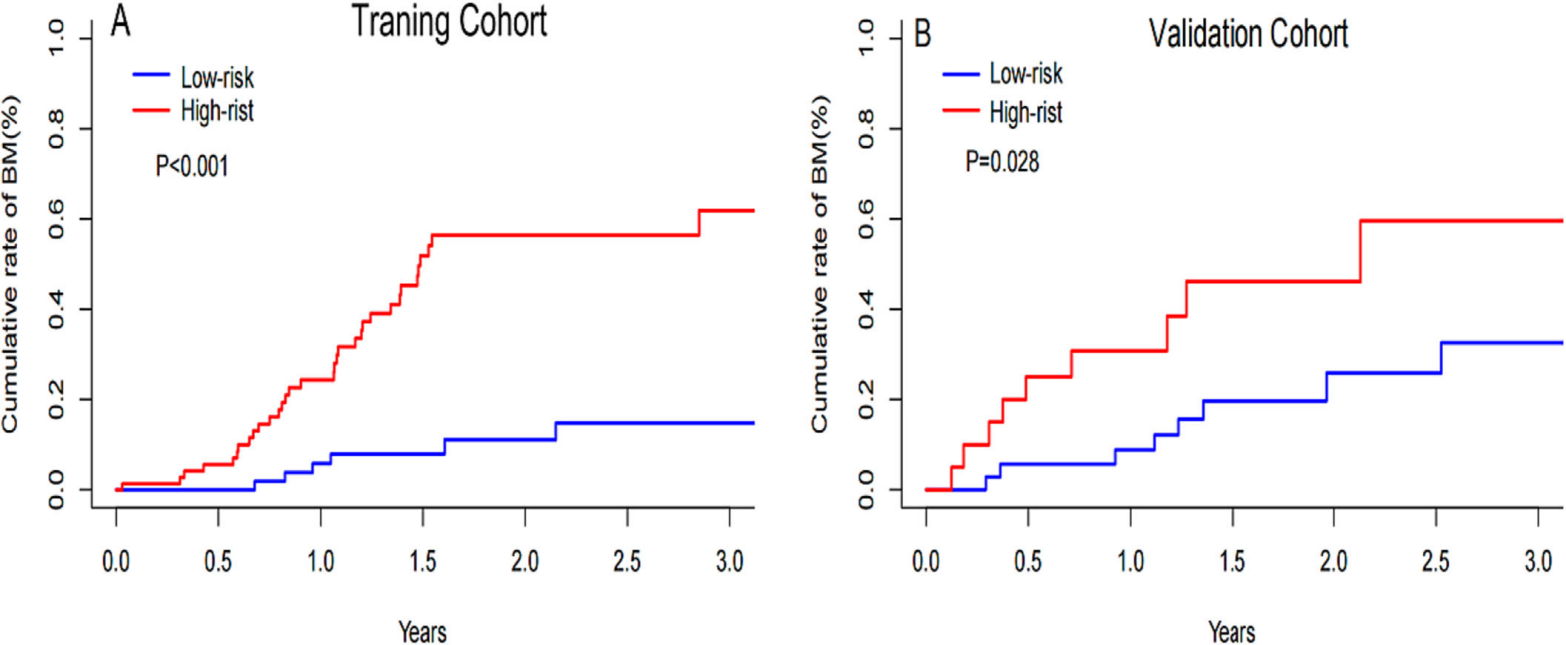
Proportion of patients with brain metastases in the training (A) and validation (B) cohorts between low‐risk and high‐risk patients.

Using a nomogram score cutoff of 277, we divided all patients into low and high‐risk groups, and it is worth noting that there were statistically significant disparities in the occurrence of BM between these two risk categories in both the training and validation cohorts. In the training cohort, the *p* value was less than 0.001, while in the validation cohort, the *p* value equaled 0.028, as illustrated in Figure 6A,B, respectively.

### 4.5 Survival analysis of OS

In Table S2, the results of univariate analysis showed a correlation between several factors and worse OS in the patient cohort. These factors include the presence of BM (*p* = 0.009), TNM stage III (*p* ≤ 0.001), ChT cycles of ≥4 (*p* = 0.014), female gender (*p* = 0.005), Ki‐67 expression ≥80%+ (*p* = 0.013), pretreatment NSE levels ≥15.4 ng/ml (*p* = 0.008), pretreatment serum LDH levels ≥200 IU/L (*p* ≤ 0.001), pretreatment CEA levels ≥10 ng/ml (*p* = 0.012), and pretreatment HGB levels ≥120 g/L (*p* = 0.092).

Subsequently, in a multivariable Cox analysis, certain factors emerged as significant independent prognostic indicators for OS. These included TNM stage (III vs. II, HR = 2.13, 95% CI = 1.18–3.85, *p* = 0.013), the presence of BM (Yes vs. No, HR = 3.56, 95% CI = 1.73–13.57, *p* = 0.043), the number of chemotherapy cycles (≥4 vs. <4, HR = 0.53, 95% CI = 0.33–0.84, *P* = 0.006), Ki‐67 expression level (<80% + vs. ≥80%+, HR = 0.56, 95% CI = 0.36–0.87, *P* = 0.010) and pretreatment LDH levels (≥200 IU/L vs. <200 IU/L, HR = 1.79, 95% CI = 1.15–2.79, *P* = 0.009). These significant factors are detailed in Supplementary Table S2.

## 5 DISCUSSION

Herein, a new nomogram was developed to predict the presence of BM in patients with LS‐SCLC who did not undergo PCI. A total of 194 cases were included, and five important prognostic factors representing pretreatment serological, immunohistochemical, staging and treatment information, that is, TNM stage, chemotherapy cycles, pretreatment LDH and HGB, and the expression level of Ki‐67, were identified by multivariate analysis. We developed a nomogram based on these factors to forecast the likelihood of BM at 2 and 3 years for LS‐SCLC patients who did not receive PCI. This nomogram demonstrated high accuracy and reliability with good clinical applicability according to the AUC (0.870) of the 2‐year BM risk and AUC (0.897) of the 3‐year BM risk in the training cohort and AUC (0.828) of the 2‐year BM risk and AUC (0.789) of the 3‐year BM risk in the validation cohort, as well as validation of discrimination and calibration.

Whether PCI can improve OS in patients with limited‐stage SCLC remains a subject of ongoing debate and controversy. For this question, some large phase III studies (NCT04155034, NCT04829708 and NCT04790253) are being constantly carried out. The results are expected within 5 years from some of the above studies. Regardless of the results, it remains of great importance to find out patients with LS‐SCLC at a low risk of BM to avoid PCI; patients having undergone PCI were not included in the prediction model of BM in this study, mainly due to the great interference of PCI with the occurrence of BM, which would obviously weaken the predictive efficacy of other factors. In addition, patients with stage I disease in our study were also excluded, mainly considering their extremely low incidence (12%) of BM,^13^ and PCI is not recommended by current National Comprehensive Cancer Network (NCCN) guidelines for patients with stage I disease after radical surgery.

At present, there are many risk factors for BM of SCLC reported worldwide, such as LDH,^14^ CEA,^15^ gastrin releasing peptide precursor (ProGRP)^16^ and PLT,^17^, ^18^ HGB^18^, neutrophil to lymphocyte ratio (NLR),^19^, ^20^, ^21^ monocyte–lymphocyte ratio (MLR),^21^ prognostic‐nutrition index (PNI),^21^ TNM stages,^15^, ^16^, ^18^, ^19^, ^22^, ^23^, ^24^, ^25^, ^26^, ^27^ lymphovascular invasion or not,^23^ radical surgery or not,^22^ concurrent chemotherapy (CCRT) or not,^21^ response to chemotherapy,^28^ weight loss,^28^ time to thoracic radiotherapy,^16^, ^19^ chemotherapy cycles,^16^, ^19^ dose of thoracic radiotherapy,^29^ size of primary tumour,^25^ segmentation method of thoracic radiotherapy,^26^ age,^27^ gender,^18^, ^27^ and so forth. However, only three of these studies established a predictive model for BM in SCLC; it is expected that prediction models for these factors of high risk of BM can be subsequently established and verified both internally and externally. The predictive risk model in stage III SCLC patients without PCI developed by Qiu et al.^21^ identified concurrent chemoradiotherapy (CCRT), lymphocyte‐to‐monocyte ratio (MLR), neutrophil‐to‐lymphocyte ratio (NLR) and the prognostic nutritional index (PNI) as independent prognostic factors for BM. Their nomogram demonstrated high accuracy in predicting the risk of BM, with a concordance index (C‐index) of 0.73, underlining its effectiveness as a predictive tool for assessing BM risk in this patient population.

In this study, our findings support the notion that patients with TNM stage III, fewer than four chemotherapy cycles and elevated pretreatment LDH (≥200 IU/L) face an increased risk of developing BM. These results echo the recent study conducted by Hou et al.,^16^ where they similarly observed significant associations between TNM stage (III vs. I to II), LDH levels (high versus low) and chemotherapy cycles (≥4 vs. <4) with the occurrence of BM, further reinforcing the consistency and robustness of these factors in predicting BM risk. In addition, their study also demonstrated that ProGRP, time of radiotherapy and lymphocyte monocyte ratio (LMR) were independent prognostic factors for BM. Meanwhile, for the assessment of individualized BM risk, the proposed nomogram showed reliable performance.

The preferred first‐line treatment for SCLC patients is still platinum‐based chemotherapy, such as cisplatin or carboplatin in combination with etoposide, in principle for four to six cycles.^30^ With this chemotherapy regimen, the complete response rate can exceed 20%, while the treatment‐related mortality rate can be kept below 5%.^3^ Two related studies have both shown that <4 or ≤4 cycles of chemotherapy are independent risk factors for BM, suggesting that a lower number of ChT cycles may not be able to kill micro‐metastases.^15^, ^19^ Of course, the duration of chemotherapy may also affect changes in patients' HGB and LDH levels, and the role it plays is complex and requires further research to confirm.

As for LDH, it is a protein related to tumour metabolism and can be applied for the detection of malignancies in serum. Specifically, LDH is the key enzyme in glycolytic metabolism that stimulates the interconversion of pyruvate to lactate. In addition, several investigators found that LDH levels are elevated during cell malignant transformation in various types of cancer, including SCLC.^30^ In patients who developed BM, serum LDH levels were inversely correlated with OS.^31^ Although there have been no reports on the association between LDH and brain metastasis in SCLC, some researchers have found that elevated LDH is often associated with poor survival in SCLC.^30^, ^32^, ^33^ LDH has been reported to promote tumour metastasis by participating in tumour angiogenesis and immune escape.^30^ However, LDH levels are also affected by a variety of factors such as chemotherapy and inflammation. Whether it can be used as a predictor of BM in SCLC patients remains to be investigated.

Li et al.^18^ identified T‐ and N‐stage, HGB, NSE and CEA as risk factors and incorporated these into the model, with a C‐index of 0.818. Our and other studies have shown that hypohemoglobinaemia is associated with a higher incidence of BM, which may be related to the consistent hypoxia in tumours caused by hypoalbuminemia that makes BM more likely to occur.^34^, ^35^, ^36^ Studies also had shown that sustained tumour hypoxia can additionally enhance malignant progression and may increase aggressiveness through clonal selection and genome changes.^35^ However, HGB is not specific as a prognostic factor because the occurrence of anaemia in cancer patients is related to multiple factors: cancer‐related (such as bone marrow infiltration, haemorrhage), treatment‐related (such as nephrotoxicity) and patient‐related (such as malnutrition).^37^ No relevant studies have been found to demonstrate the intrinsic association of anaemia with brain metastasis in SCLC. Therefore, further basic researches are needed to investigate whether HGB can be used as a predictor for brain metastasis.

Ki‐67 is a protein that is expressed in the nucleus of proliferating mammalian cells. Its function remains unclear, although it is widely used in the study of cancer histopathology.^38^ In studies of lung cancer,^39^ Ki‐67 was found to be a proliferation marker of malignant tumours. Based on tumour cellularity, Ki‐67 positivity and proliferating cell nuclear antigen (PCNA), together with clinical stage and histological differentiation, provided additional useful information for predicting lung cancer evolution and prognosis. In the study by Cai Q et al.,^40^ Ki‐67 antigen showed a significant correlation with survival of SCLC. However, no studies reported the association between Ki‐67 and BM in SCLC. This study firstly found that Ki‐67 ≥ 80%+ was an independent risk factor for BM.

In summary, after the above discussion, we preliminarily speculate that TNM stage and ChT cycle may be the main factors in predicting BM, while Ki‐67 expression and serum LDH level are factors that need to be confirmed. In addition, HGB levels are more affected by other factors and may not actually be a predictor. Of course, further research is still needed to confirm whether they are truly independent predictors.

In addition, it is essential to acknowledge the limitations of this study. First, it is a small‐scale, single‐centre, retrospective investigation, lacking the robustness of a larger, randomized controlled trial with its inherent advantages. Moreover, due to constraints related to technology and financial resources, the study did not incorporate radiomics or pathomics into the analysis. Additionally, the nomogram models developed were not externally validated in an independent cohort, warranting further validation to enhance their reliability and generalizability.

## 6 CONCLUSION

To summarize, our study revealed that TNM stage, ChT cycles, Ki‐67 expression, pretreatment LDH and HGB levels were linked to the risk of BM in LS‐SCLC patients. The nomogram‐based model shows promise in assisting clinicians with BM risk stratification and potentially reducing unnecessary PCI utilization. However, it is crucial to note that this model remains exploratory and requires validation in external datasets to confirm its reliability and applicability beyond our study cohort.

## AUTHOR CONTRIBUTIONS

Jiayi Guo and Jianjiang Liu wrote the main text of the manuscript and prepared the figures and tables. Wanli Ye, Jun Xu, Wangyan Zhong, Xiaoyu Zhang, Hang Yuan, Hao Shi, Ting Li, Yibing Xu and Jiwei Mao collected clinical data. Bin Shen and Dongping Wu were responsible for research design. All authors reviewed the manuscript.

## CONFLICT OF INTEREST STATEMENT

The authors have disclosed that they have no competing interest to declare.

## ETHICS STATEMENT

This research received ethical approval from the Ethics Committee of Shaoxing People's Hospital and was conducted in accordance with the principles outlined in the Declaration of Helsinki. All authors have provided their consent for the publication of this article.

## Supporting information


**Table S1.** Clinical characteristics of patients in the training and validation cohorts.
**Table S2.** Univariate and multivariate analysis predicting overall survival (OS) of the all patients without prophylactic cranial irradiation (PCI).

## Data Availability

The data that support the findings of this study are available on request from the corresponding author. The data are not publicly available due to privacy or ethical restrictions.
