# Supplementary material for: Construction of brain metastasis prediction model in limited stage small cell lung cancer patients without prophylactic cranial irradiation
Source: Clin Respir J. 2024 Jan 15;18(1):e13730. doi: 10.1111/crj.13730 (PMC10790059; doi:10.1111/crj.13730)
Supplement: Supplementary file 1 — Table S1. Clinical characteristics of patients in the training and validation cohorts. Table S2. Univariate and multivariate analysis predicting overall survival (OS) of the all patients without prophylactic cranial irradiation (PCI). [file CRJ-18-e13730-s001.docx]

**ABBREVIATIONS**

Small cell lung cancer (SCLC); prophylactic cranial irradiation (PCI); brain metastasis (BM); limited stage small-cell lung cancer (LS-SCLC);hazard ratio (HR); confidence interval (CI); complete response (CR); chemotherapy cycles (ChT cycles); neuron specific enolase (NSE); serum lactate dehydrogenase (LDH); platelet (PLT); hemoglobin (HGB); carcinoembryonic antigen (CEA); receiver operating characteristic (ROC); area under the ROC curve (AUC); Decision curve analysis (DCA); magnetic resonance imaging (MRI); overall survival (OS); 18F-fluorodeoxyglucose (18F-FDG PET); computed tomography (CT); Alternate Joint communication Center (AJCC); emission computed tomography (ECT); Response Evaluation Criteria in Solid Tumors (RECIST); South West Oncology Group (SWOG); extensive stage small-cell lung cancer (ES-SCLC); European Organization for Research and Treatment of Cancer (EORTC); National Comprehensive Cancer Network (NCCN); no appearance of BM (No BM); gastrin releasing peptide precursor (ProGRP); neutrophil to lymphocyte ratio (NLR); monocyte–lymphocyte ratio (MLR); prognostic-nutrition index (PNI); concurrent chemotherapy (CCRT); Eastern Cooperative Oncology Group Performance Status (ECOG-PS); Proliferating Cell Nuclear Antigen (PCNA).

| **Supplementary Table S1. Clinical characteristics of patients in the training and validation cohorts.** | | | | | |
| --- | --- | --- | --- | --- | --- |
| **Variable** | **Total**  **(n = 194)** | **Training**  **(n = 136)** | **Validation**  **(n = 58)** | **Statistic** | **P*** |
| Gender, n (%) |  |  |  | χ²=0.015 | 0.902 |
| Male | 135 (69.59) | 95 (69.85) | 40 (68.97) |  |  |
| Female | 59 (30.41) | 41 (30.15) | 18 (31.03) |  |  |
| Age, n (%) |  |  |  | χ²=0.187 | 0.665 |
| <60 | 44 (22.68) | 32 (23.53) | 12 (20.69) |  |  |
| ≥60 | 150 (77.32) | 104 (76.47) | 46 (79.31) |  |  |
| Smoking index*, n (%) |  |  |  | χ²=2.358 | 0.308 |
| 0 | 76 (39.18) | 58 (42.65) | 18 (31.03) |  |  |
| 1-800 | 78 (40.21) | 51 (37.50) | 27 (46.55) |  |  |
| >800 | 40 (20.62) | 27 (19.85) | 13 (22.41) |  |  |
| ECOG-PS*, n (%) |  |  |  | χ²=2.206 | 0.137 |
| 0-1 | 168 (86.6) | 121 (88.97) | 47 (81.03) |  |  |
| ≥2 | 26 (13.4) | 15 (11.03) | 11 (18.97) |  |  |
| TNM stage, n (%) |  |  |  | χ²=0.008 | 0.929 |
| II | 56 (28.87) | 39 (28.68) | 17(29.31) |  |  |
| III | 138 (71.13) | 97 (71.32) | 41 (70.69) |  |  |
| ChT* cycles, n (%) |  |  |  | χ²=7.814 | 0.101 |
| <4 | 118 (60.82) | 100 (73.53) | 18 (31.03) |  |  |
| ≥4 | 76 (39.18) | 36 (26.47) | 40 (68.97) |  |  |
| Radiotherapy, n (%) |  |  |  | χ²=0.067 | 0.796 |
| No | 111 (57.22) | 77 (56.62) | 34 (58.62) |  |  |
| Yes | 83 (42.78) | 59 (43.38) | 24 (41.38) |  |  |
| Concurrent ChT, n (%) |  |  |  | χ²=0.509 | 0.476 |
| No | 147 (75.77) | 105 (77.21) | 42 (72.41) |  |  |
| Yes | 47 (24.23) | 31 (22.79) | 16 (27.59) |  |  |
| Treatment Response, n (%) |  |  |  | χ²=1.528 | 0.561 |
| CR* | 68 (35.05) | 32 (23.53) | 36 (62.07) |  |  |
| No CR | 126 (64.95) | 104 (76.47) | 22 (37.93) |  |  |
| TTF-1*, n (%) |  |  |  | χ²=0.000 | 0.983 |
| - | 60 (30.93) | 42 (30.88) | 18 (31.03) |  |  |
| + | 134 (69.07) | 94 (69.12) | 40 (68.97) |  |  |
| Ki-67, n (%) |  |  |  | χ²=3.174 | 0.071 |
| <80+ | 86 (44.33) | 72 (52.94) | 14 (24.14) |  |  |
| ≥80+ | 108 (55.67) | 64 (47.06) | 44 (75.86) |  |  |
| Pretreatment CEA*, n (%) |  |  |  | χ²=0.189 | 0.663 |
| <10ng/ml | 138 (71.13) | 98 (72.06) | 40 (68.97) |  |  |
| ≥10ng/ml | 56 (28.87) | 38 (27.94) | 18 (31.03) |  |  |
| Pretreatment HGB*, n (%) |  |  |  | χ²=1.818 | 0.178 |
| >120g/L | 96 (49.48) | 63 (46.32) | 33 (56.90) |  |  |
| ≤120g/L | 98 (50.52) | 73 (53.68) | 25 (43.10) |  |  |
| Pretreatment PLT*, n (%) |  |  |  | χ²=0.083 | 0.773 |
| <280*10^9^/L | 64 (32.99) | 44 (32.35) | 20 (34.48) |  |  |
| ≥280*10^9^/L | 130 (67.01) | 92 (67.65) | 38 (65.52) |  |  |
| Pretreatment LDH*, n (%) |  |  |  | χ²=0.614 | 0.433 |
| <200IU/L | 94 (48.45) | 62 (45.59) | 32 (51.72) |  |  |
| ≥200IU/L | 100 (51.55) | 74 (54.41) | 26 (48.28) |  |  |
| Pretreatment NSE*, n (%) |  |  |  | χ²=3.079 | 0.053 |
| <15.4ng/ml | 153 (78.87) | 102 (75.00) | 51 (87.93) |  |  |
| ≥15.4ng/ml | 41 (21.13) | 34 (25.00) | 7 (12.07) |  |  |

P=Pearson’s χ^2^ test was used to calculate the p-value, Smoking index=Number of cigarettes smoked per day × years of smoking, ECOG-PS=Eastern Cooperative Oncology Group performance status, ChT=chemotherapy, CR=complete response, TTF-1=thyroid transcription factor-1, CEA=carcinoembryonic antigen, HGB=hemoglobin, PLT=platelet, LDH=serum lactate dehydrogenase, NSE=neuron specific enolase.

**Supplementary Table S2. Univariate and multivariate analysis predicting overall survival (OS) of the all patients without prophylactic cranial irradiation (PCI).**

| **Variables** | **Univariate analysis** | | | **Multivariate analysis** | | |
| --- | --- | --- | --- | --- | --- | --- |
|  | **HR*** | **95% CI*** | **P** | **HR** | **95% CI** | **P** |
| BM (Yes vs. No) | 5.37 | 1.53-18.86 | 0.009 | 3.56 | 1.73-13.57 | 0.043 |
| TNM Stage (III vs. II) | 2.70 | 1.59-4.45 | <.001 | 2.13 | 1.18-3.85 | 0.013 |
| ChT cycles (≥4 vs. <4) | 0.59 | 0.38-0.90 | 0.014 | 0.53 | 0.33-0.84 | 0.006 |
| Ki-67 (<80%+ vs. ≥80%+) | 0.60 | 0.40-0.90 | 0.013 | 0.56 | 0.36-0.87 | 0.010 |
| Pretreatment LDH  (≥200IU/L vs. <200IU/L) | 2.01 | 1.35-3.00 | <.001 | 1.79 | 1.15-2.79 | 0.009 |
| Gender (female vs. male) | 0.52 | 0.33-0.82 | 0.005 | 0.64 | 0.38-1.10 | 0.108 |
| Smoking index |  |  |  |  |  |  |
| (1-800 vs. 0) | 1.71 | 1.09-2.67 | 0.019 | 1.31 | 0.77-2.24 | 0.323 |
| (＞800 vs. 0) | 1.51 | 0.87-2.62 | 0.145 | 1.03 | 0.58-1.86 | 0.910 |
| Pretreatment CEA  (≥10ng/ml vs. <10ng/ml) | 1.69 | 1.12-2.56 | 0.012 | 1.47 | 0.96-2.26 | 0.075 |
| Pretreatment NSE  (≥15.4ng/ml vs. <15.4ng/ml) | 1.79 | 1.16-2.78 | 0.008 | 1.18 | 0.72-1.92 | 0.509 |
| Pretreatment HGB  (≥120g/L vs. <120g/L) | 1.38 | 1.16-2.04 | 0.108 |  |  |  |
| Age (≥60 vs. <60) | 0.86 | 0.54-1.38 | 0.538 |  |  |  |
| Pretreatment PLT  (≥280*109/L vs. <280*109/L) | 0.89 | 0.58-1.35 | 0.574 |  |  |  |
| Treatment Response  (No CR vs. CR) | 1.20 | 0.80- 1.81 | 0.371 |  |  |  |
| ECOG-PS (0-1 vs. ≥2) | 0.94 | 0.54-1.63 | 0.828 |  |  |  |
| Radiotherapy (Yes vs. NO) | 1.14 | 0.77-1.69 | 0.524 |  |  |  |
| Concurrent ChT (Yes vs. No) | 1.26 | 0.80-1.97 | 0.320 |  |  |  |
| TTF-1 (+ vs. -) | 1.07 | 0.70-1.63 | 0.753 |  |  |  |

HR=hazard ratio; CI=confidence interval.
